# Bacterial Ventures into Multicellularity: Collectivism through Individuality

**DOI:** 10.1371/journal.pbio.1002162

**Published:** 2015-06-03

**Authors:** Simon van Vliet, Martin Ackermann

**Affiliations:** 1 Institute of Biogeochemistry and Pollutant Dynamics, Department of Environmental Systems Science, ETH Zurich, Zurich, Switzerland; 2 Department of Environmental Microbiology, Eawag, Dübendorf, Switzerland; 3 Life Science Zurich Graduate School, PhD program in Systems Biology, ETH Zurich, Zurich, Switzerland

## Abstract

Multicellular eukaryotes can perform functions that exceed the possibilities of an individual cell. These functions emerge through interactions between differentiated cells that are precisely arranged in space. Bacteria also form multicellular collectives that consist of differentiated but genetically identical cells. How does the functionality of these collectives depend on the spatial arrangement of the differentiated bacteria? In a previous issue of *PLOS Biology*, van Gestel and colleagues reported an elegant example of how the spatial arrangement of differentiated cells gives rise to collective behavior in *Bacillus subtilus* colonies, further demonstrating the similarity of bacterial collectives to higher multicellular organisms.

## Abstract 2

This Primer discusses how interactions between differentiated and spatially organized cells give rise to an elegant example of collective behavior in *Bacillus subtilis* colonies. Read the Research Article.

Introductory textbooks tend to depict bacteria as rather primitive and simple life forms: the billions of cells in a population are all supposedly performing the exact same processes, independent of each other. According to this perspective, the properties of the population are thus nothing more than the sum of the properties of the individual cells. A brief look at the recent literature shows that life at the micro scale is much more complex and far more interesting. Even though cells in a population share the same genetic material and are exposed to similar environmental signals, they are individuals: they can greatly differ from each other in their properties and behaviors [1,2].

One source of such phenotypic variation is that individual cells experience different microenvironments and regulate their genes in response. However, and intriguingly, phenotypic differences can also arise in the absence of environmental variation [3]. The stochastic nature of biochemical reactions makes variation between individuals unavoidable: reaction rates in cells will fluctuate because of the typical small number of the molecules involved, leading to slight differences in the molecular composition between individual cells [4]. While cells cannot prevent fluctuations from occurring, the effect of these extracellular and intracellular perturbations on a cell’s phenotype can be controlled by changing the biochemical properties of molecules or the architecture of gene regulatory networks [4,5]. The degree of phenotypic variation could thus evolve in response to natural selection. This raises the question of whether the high degree of phenotypic variation observed in some traits could offer benefits to the bacteria [5].

One potential benefit of phenotypic variation is bet hedging. Bet hedging refers to a situation in which a fraction of the cells express alternative programs, which typically reduce growth in the current conditions but at the same time allow for increased growth or survival when the environment abruptly changes [6–8]. Another potential benefit can arise through the division of labor: phenotypic variation can lead to the formation of interacting subpopulations that specialize in complementary tasks [9]. As a result, the population as a whole can perform existing functions more efficiently or attain new functionality [10]. Division of labor enables groups of bacteria to engage in two tasks that are incompatible with each other but that are both required to attain a certain biological function.

One of the most famous examples of division of labor in bacteria is the specialization of multicellular cyanobacteria into photosynthesizing and nitrogen-fixing subpopulations [11]. Here, the driving force behind the division of labor is the biochemical incompatibility between photosynthesis and nitrogen fixation, as the oxygen produced during photosynthesis permanently damages the enzymes involved in nitrogen fixation [12]. Other examples include the division of labor between two subpopulations of *Salmonella* Typhimurium (Tm) during infections [9] and the formation of multicellular fruiting bodies in *Myxococcus xanthus* [13]. Division of labor is not restricted to interactions between only two subpopulations; for example, the soil-dwelling bacteria *Bacillus subtilis* can differentiate into at least five different cell types [14]. Multiple types can simultaneously be present in *Bacillus* biofilms and colonies, each contributing different essential tasks [14,15].

An important question is whether a successful division of labor requires the different subpopulations to coordinate their behavior and spatial arrangement. For some systems, it turns out that spatial coordination is not required. For example, the division of labor in clonal groups of *Salmonella* Tm does not require that the two cell types are spatially arranged in a particular way [9]. In other systems, spatial coordination between the different cell types seems to be beneficial. For example, differentiation into nitrogen-fixing and photosynthetic cells in multicellular cyanobacteria is spatially regulated in a way that facilitates sharing of nitrogen and carbon [16]. In general, when cell differentiation is combined with coordination of behavior between cells, this can allow for the development of complex, group-level behaviors that cannot easily be deduced from the behavior of individual cells [17–20]. In these cases, a population can no longer be treated as an assembly of independent individuals but must be seen as a union that together shows collective behavior.

The study by van Gestel et al. [21] in a previous issue of *PLOS Biology* offers an exciting perspective on how collective behavior can arise from processes operating at the level of single cells. Van Gestel and colleagues [21] analyzed how groups of *B*. *subtilis* cells migrate across solid surfaces in a process known as sliding motility. The authors found that migration requires both individuality—the expression of different phenotypes in clonal populations—and spatial coordination between cells. Migration depends critically on the presence of two cell types: surfactin-producing cells, which excrete a surfactant that reduce surface tension, and matrix-producing cells, which excrete extracellular polysaccharides and proteins that form a connective extracellular matrix (Fig 1B) [14]. These two cell types are not randomly distributed across the bacterial group but are rather spatially organized (Fig 1C). The matrix-producing cells form bundles of interconnected and highly aligned cells, which the authors refer to as “van Gogh” bundles. The surfactant producing cells are not present in the van Gogh bundle but are essential for the formation of the bundles [21].

**Fig 1:**
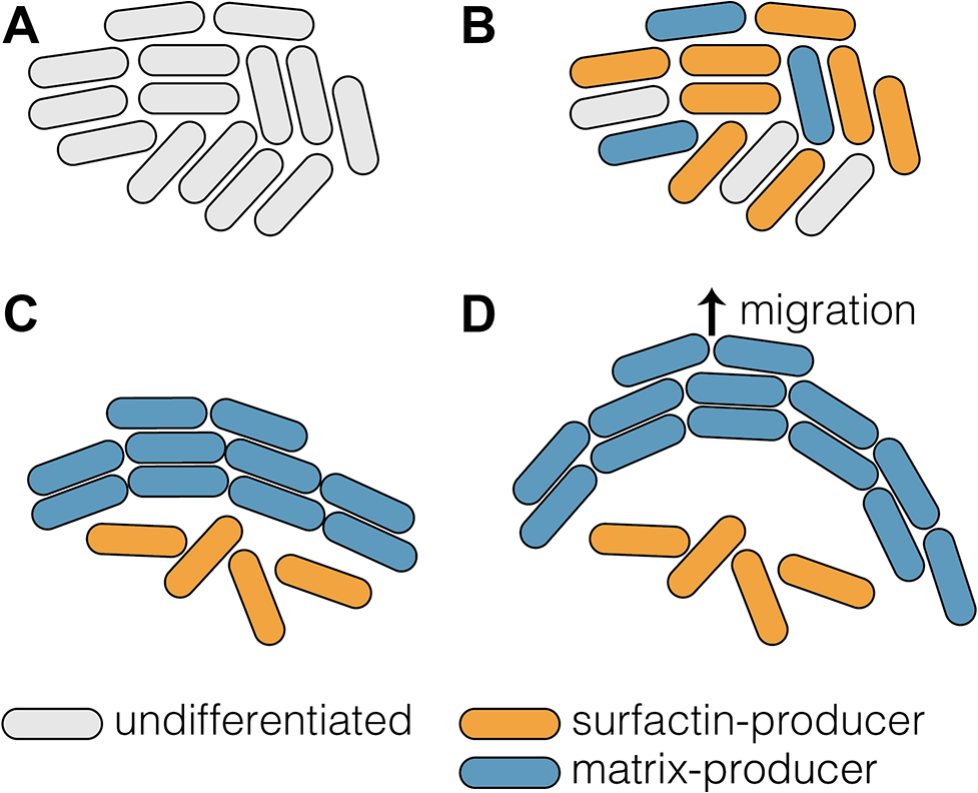
Collective behavior through the spatial organization of differentiated cells. (A) Initially cells form a homogenous population. (B) Differentiation: cells start to differentiate into surfactin- (orange) and matrix- (blue) producing cells. The two cell types perform two complementary and essential tasks, resulting in a division of labor. (C) Spatial organization: the matrix-producing cells form van Gogh bundles, consisting of highly aligned and interconnected cells. Surfactin-producing cells are excluded from the bundles and have no particular spatial arrangement. (D) Collective behavior: growth of cells in the van Gogh bundles leads to buckling of these bundles, resulting in colony expansion. The buckling and resulting expansion depend critically on the presence of the two cell types and on their spatial arrangement.

The ability to migrate is a collective behavior that can be linked to the biophysical properties of the multicellular van Gogh bundles. The growth of cells in these bundles causes them to buckle, which in turn drives colony migration (Fig 1D) [21]. This is a clear example of an emergent (group-level) phenotype: the buckling of the van Gogh bundles and the resulting colony motility cannot easily be deduced from properties of individual cells. Rather, to understand colony migration we have to understand the interactions between the two cells type as well as their spatial organization. Building on a rich body of work on the regulation of gene expression and cellular differentiation in *Bacillus* [14], van Gestel et al. [21] are able to show how these molecular mechanisms lead to the formation of specialized cell types that, through coordinated spatial arrangement, provide the group the ability to move (Fig 1). The study thus uniquely bridges the gap between molecular mechanisms and collective behavior in bacterial multicellularity.

This study raises a number of intriguing questions. A first question pertains to the molecular mechanisms underlying the spatial coordination of the two cell types. Can the spatial organization be explained based on known mechanisms of the regulation of gene expression in this organism or does the formation of these patterns depend on hitherto uncharacterized gene regulation based on spatial gradients or cell–cell interaction? A second question is about the selective forces that lead to the evolution of collective migration of this organism. The authors raise the interesting hypothesis that van Gogh bundles evolved to allow for migration. Although this explanation is very plausible, it also raises the question of how selection acting on a property at the level of the group can lead to adaptation at the individual cell level. Possible mechanisms for such selective processes have been described within the framework of multilevel selection theory. However, there are still many questions regarding how, and to what extent, multilevel selection operates in the natural world [22–24]. The system described by van Gestel and colleagues [21] offers exciting opportunities to address these questions using a highly studied and experimentally amenable model organism.

Bacterial collectives (e.g., colonies or biofilms) have been likened to multicellular organisms partly because of the presence of cell differentiation and the importance of an extracellular matrix [25,26]. Higher multicellular organisms share these properties; however, they are more than simple lumps of interconnected, differentiated cells. Rather, the functioning of multicellular organisms critically depends on the precise spatial organization of these cells [27]. Even though spatial organization has been suggested before in *B*. *subtilis* biofilms [28], there was a gap in our understanding of how spatial organization of unicellular cells can lead to group-level function. The van Gogh bundles in the article by van Gestel et al. [21] provide direct evidence on how differentiated cells can spatially organize themselves to give rise to group-level behavior. This shows once more that bacteria are not primitive “bags of chemicals” but rather are more like us “multicellulars” than we might have expected.
